# Wearable Flexible Electronics Based Cardiac Electrode for Researcher Mental Stress Detection System Using Machine Learning Models on Single Lead Electrocardiogram Signal

**DOI:** 10.3390/bios12060427

**Published:** 2022-06-17

**Authors:** Md Belal Bin Heyat, Faijan Akhtar, Syed Jafar Abbas, Mohammed Al-Sarem, Abdulrahman Alqarafi, Antony Stalin, Rashid Abbasi, Abdullah Y. Muaad, Dakun Lai, Kaishun Wu

**Affiliations:** 1 IoT Research Center, College of Computer Science and Software Engineering, Shenzhen University, Shenzhen 518060, China; belalheyat@gmail.com; 2 School of Computer Science and Engineering, University of Electronic Science and Technology of China, Chengdu 610056, China; 201914080110@std.uestc.edu.cn; 3 Faculty of Management, Vancouver Island University, Nanaimo, BC V9R5S5, Canada; jafar1987abbas@stumail.viu.ca; 4 College of Computer Science and Engineering, Taibah University, Medina 42353, Saudi Arabia; asalqarafi@taibahu.edu.sa; 5 Department of Computer Science, University of Sheba Province, Marib, Yemen; 6 Institute of Fundamental and Frontier Sciences, University of Electronic Science and Technology of China, Chengdu 610054, China; a.staanlin@gmail.com; 7 School of Electrical Engineering, Anhui Polytechnic University, Wuhu 241000, China; rashid.abbasi@uestc.edu.cn; 8 Department of Studies in Computer Science, University of Mysore, Mysore 570005, Karnataka, India; abdullahmuaad9@gmail.com; 9 IT Department, Sana’a Community College, Sana’a 5695, Yemen; 10 School of Electronic Science and Engineering, University of Electronic Science and Technology of China, Chengdu 610054, China

**Keywords:** diagnosis, decision tree, electrode, flexible electronics, machine learning, mitochondria, oxidative stress, overwork, stress, smart device

## Abstract

In the modern world, wearable smart devices are continuously used to monitor people’s health. This study aims to develop an automatic mental stress detection system for researchers based on Electrocardiogram (ECG) signals from smart T-shirts using machine learning classifiers. We used 20 subjects, including 10 from mental stress (after twelve hours of continuous work in the laboratory) and 10 from normal (after completing the sleep or without any work). We also applied three scoring techniques: Chalder Fatigue Scale (CFS), Specific Fatigue Scale (SFS), Depression, Anxiety, and Stress Scale (DASS), to confirm the mental stress. The total duration of ECG recording was 1800 min, including 1200 min during mental stress and 600 min during normal. We calculated two types of features, such as demographic and extracted by ECG signal. In addition, we used Decision Tree (DT), Naive Bayes (NB), Random Forest (RF), and Logistic Regression (LR) to classify the intra-subject (mental stress and normal) and inter-subject classification. The DT leave-one-out model has better performance in terms of recall (93.30%), specificity (96.70%), precision (94.40%), accuracy (93.30%), and F1 (93.50%) in the intra-subject classification. Additionally, The classification accuracy of the system in classifying inter-subjects is 94.10% when using a DT classifier. However, our findings suggest that the wearable smart T-shirt based on the DT classifier may be used in big data applications and health monitoring. Mental stress can lead to mitochondrial dysfunction, oxidative stress, blood pressure, cardiovascular disease, and various health problems. Therefore, real-time ECG signals help assess cardiovascular and related risk factors in the initial stage based on machine learning techniques.

## 1. Introduction 

Long working times are an essential issue in the world [1,2]. It leads to many disorders such as mental disorders, cerebrovascular/cardiovascular diseases, diabetes, and cancer. According to China Daily (http://www.chinadaily.com.cn/china/2016-12/11/content_27635578.htm accessed on 20 November 2021) (http://www.chinadaily.com.cn/opinion/2012-10/31/content_15859379.htm accessed on 20 November 2021) approximately six hundred thousand Chinese people die due to overwork. The International Labor Organization (http://www.ilo.org/global/topics/safety-and-health-at-work/lang--de/index.htm accessed on 20 November 2021) reported that about 2.78 million employees die yearly because of overwork. China Radio International stated that approximately 1600 people die every day because of the long-time spans in the workplace (https://www.beaconjournal.com/article/20140629/news/306299492 accessed on 20 November 2021). In Japan, 368 people have committed suicide in 5 years due to overwork. According to the Japanese government, more than 200 people died due to long working times (https://www.nupoliticalreview.com/2020/07/15/japans-vicious-death-by-overwork-cycle/ accessed on 20 November 2021) [3,4]. It is tough to calculate overwork based on the number of working hours. So, mental stress is an excellent way to detect overwork. It is a type of mental tiredness due to overwork or continuous work. Overwork is a significant source of shortening human life.

Stress is a kind of symptom of underlying sicknesses. The leading causes of stress are anemia, antihistamines, congestive heart failure, depression, hypothyroidism, insomnia, muscle exertion, narcolepsy, obesity, and tuberculosis. The main symptoms of stress are brittle hair, dry skin, polydipsia, polyuria, shortness of breath, and tiredness. It is divided into two parts such as physical stress and mental stress. Physical stress is exertion due to physical work such as lifting, playing, and running continuously for a long time. 

Mental stress is prevalent at this time, related to deteriorating performance on cognitive tasks [5,6]. Mental stress also affects accidents and injuries associated with human life, such as reducing work output, making decisions, and sleeping problems. It is speculated that acute mental stress could lead to atrial and ventricular arrhythmias. Electrical signal processing techniques such as the Electrocardiogram (ECG) explain stress-induced arrhythmias through electrophysiological mechanisms. In general, stress affects the significant components of the averaged ECG signals, and the changes that are repeated long-term by stressors could lead to arrhythmias [7]. The long-term stress response of the human body releases a high level of cortisol hormone, which can increase blood pressure, blood glucose levels, cholesterol levels, and triglycerides, which are the significant risk factors for heart problems. In addition, the increased level of cortisol causes stimulation of the Sympathetic Nervous System (SNS), which organizes the activities of body functions during a rapid stress response to severe circumstances; similarly, the Parasympathetic Nervous System (PNS) has been activated during the non-stress time and normalizes body functions, including cardiac function [8,9]. The heart is the most important organ with high oxygen consumption, which is very sensitive to oxidative reactions and prone to oxidative stress [10]. There are many reasons for the increase in the production of Reactive Oxygen Species (ROS). Besides, the aging factor, mental stress, and other related factors could affect the functions of mitochondria. 

On the other hand, specific molecules resembling bacterial molecules may be released by mitochondria due to disruption or damage. Based on this, our immune system may treat them as foreign bodies and trigger a harmful inflammatory response against our own cells. Stress-induced mitochondrial dysfunction may alter the dynamics of cardiac function due to changes in the electrophysiological substrate and increase susceptibility to arrhythmias [11]. Mitochondrial dysfunction or damage also increases ROS production, which reduces the ability of Adenosine Triphosphate (ATP) production due to the disruption of mitochondrial membrane potentials and altered cellular redox potential. So, these kinds of excessive ROS production can lead to cardiac arrhythmias, myocardial remodeling, and cell hypertrophy [12,13]. ECG data and diagnosis can detect most cardiac arrhythmias and cardiovascular and related diseases in the early stages. Moreover, many studies have shown that ECG abnormalities are the sovereign interpreter for coronary artery disease and related problems [14,15].

Various researchers have detected mental stress using physiological signals such as Electroencephalogram (EEG) [16,17,18], Electrooculogram (EOG), ECG [19,20], and Electromyogram (EMG). Zhang et al. [21] designed convolutional and brain function-based mental stress detection using a partially directed coherence graph as a neural network classifier. Wu et al. [22] represented the pilots’ stress detection using a deep learning classifier on the EEG signal. Ahmadi et al. [23] designed a wavelet-based automatic system for mental stress detection in drivers using a Support Vector Machine (SVM) based machine learning classifier. They used eye-tracking technology to record the signals. Chen et al. [24] developed the stress model of watching television using EEG signals. Monteiro et al. [25] designed a sensor fusion-based system to recognize mental stress using physiological signals, including EEG, ECG, and EMG. Zhen et al. [26] used 15 subjects between the ages of 22 to 33 to develop a detection system of mental stress on functional near-infrared spectroscopy data. Pang et al. [27] recorded the EEG signal for 25 h and concluded that the auditory vigilance task could easily recognize mental stress. Chang et al. [28] used the wireless EMG, a thermometer, and a hand dynamometer to analyze the data and detect the mental stress of security guards. Li et al. [29] used 21 features of the single-channel EEG signal to detect mental stress. Laurent et al. [30] studied that multimodal information improved the detection of mental stress using physiological signals such as EEG, ECG, and EOG. Xiao et al. [31] suggested that using brain signals, Principal Component Analysis (PCA) and SVM classifiers easily detect mental stress in manufacturing industry workers. 

In addition, Le et al. [32] designed a Machine Learning (ML) framework to detect lung cancer. Ahamed et al. [33] reported an ecological etiquette for preparing Ag/RGO NCs using orange peel extract to treat cancer. Moreover, he also introduced the role of Zn-doping in the anticancer activity of Bi2O3 NPs [34]. Previously, some researchers suggested that ML is suitable for the automatic classification, prediction, and detection of psycho-neurological human behaviors [35,36,37,38,39]. The groups of Lai [40,41,42,43,44,45,46] and Siddiqui [47,48,49,50] have also used machine learning models to automatically detect sleep disorders such as bruxism and insomnia based on physiological signals. 

Wearable smart devices are mainly used by humans in today’s modern world. The wearable smart devices record the physiological signals, heartbeat, temperature, and time of sleeping and waking [51,52]. In the proposed study, we used a smart T-shirt to detect researchers’ mental stress based on demographic and extracted features from ECG signals. We extracted nine features from the ECG signal. As per our knowledge, it is the first time to detect the researcher’s mental stress. In addition, we used four machine learning classifiers: Decision Tree (DT), Naive Bayes (NB), Random Forest (RF), and Logistic Regression (LR), with four models as leave one out and three cross-validation folds, such as 10, 3, and 2. Additionally, we also used a DT classifier for inter-subject classification. We have developed an automatic real-time mental stress detection system using a single lead ECG signal of the wearable smart T-shirt. The main contributions of this study are:

(a) Introducing flexible dry electrodes based on wearable smart T-shirts to monitor researchers’ health.
(b) An automatic system based on extracted features from ECG signals and demographic features for the detection of researchers’ mental stress.
(c) Relationship between demographic and extracted features based on clustering technique.
(d) Comparison between different machine learning classifiers to find a suitable classification method for the automatic mental stress detection system.
(e) To achieve the average accuracy for the inter-subject (subject-wise) classification using the best performer classifier of the intra-subject (mental stress and normal) classification.

The proposed paper is organized in the standard format, such as the introduction, details of the wearable smart-shirt, materials and methods, results and discussion, and conclusion of the study. 

## 2. Wearable Smart T-Shirt

**Figure 1:**
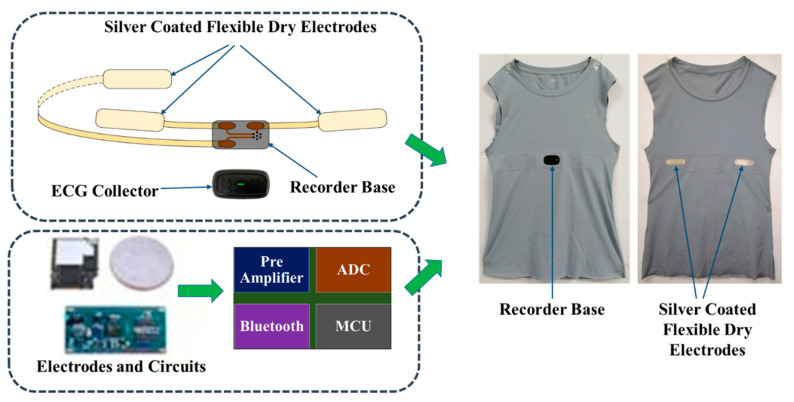
ECG acquisition system of the wearable smart T-shirt used in this study. A wearable smart T-shirt has three silver-coated dry electrodes, one smart textile, one recorder base, and one ECG collector [56,57]. The ECG signal is transferred through Bluetooth in display devices, such as smartphones, computers, laptops, and tablets.

**Figure 2:**
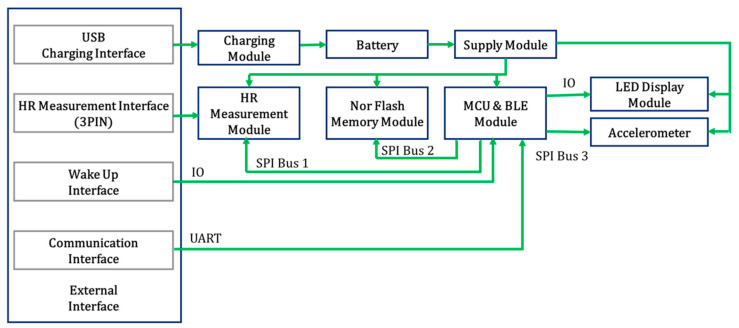
Circuit module diagram of the wearable smart T-shirt.

People’s health monitoring is a measurement challenge in the current world because people’s health is influenced due to stressful life. We used a smart T-shirt to monitor researchers’ cardiac activity. We recorded the ECG signals to develop the detection of mental stress. This device was developed by Hexin Medical Co. Ltd., Shenzhen, China. Silver-coated flexible dry electrodes with screen printing technology were used to develop this smart T-shirt. This device is capable of processing ECG signals in real-time. The processing time is less than 0.1 s for each 10 s segment with the embedded Microcontroller (MCU). It consists of a Bluetooth 4.0 model with a 2.4 GHz frequency, a 0.259-watt battery for up to 24 h of data acquisition, a smartphone, and an effective distance for data transmission of 10 m. According to the International Standards, the data packet loss rate of Bluetooth is less than 1% [53]. The sampling frequency of the ECG signal is 250 Hz. The wearable smart T-shirt works based on flexible dry electrodes and low-power ECG signals. The main function of this device is to record ECG and transfer the signal to the smartphone or other display devices. Smart clothes or textiles for the wearable system were first introduced in 1990 [54,55]. Our smart T-shirt is stationary and wearable; it has built-in textile silver-coated dry Nano electrodes. These silver-coated dry Nano electrodes are pasted on the right side, left side, and right side on the back of the chest. All electrodes are connected to the recorder base. The ECG recorder is connected to the base during the recording of the ECG signal. The recorder has a Pre-amplifier, an Analog to Digital Converter (ADC), a Nordic MCU model nRF528xx, and Bluetooth for recording, amplification, conversion, data acquisition, and transmission of the recorded ECG signal to display devices such as smartphones, laptops, and so on. All operations are performed by line-by-line command and saving the time for data acquisition. The system for ECG data acquisition by a wearable smart T-shirt is shown in Figure 1 [56,57] and the circuit module diagram of the device are mentioned in Figure 2. 

## 3. Materials and Methods

**Figure 3:**
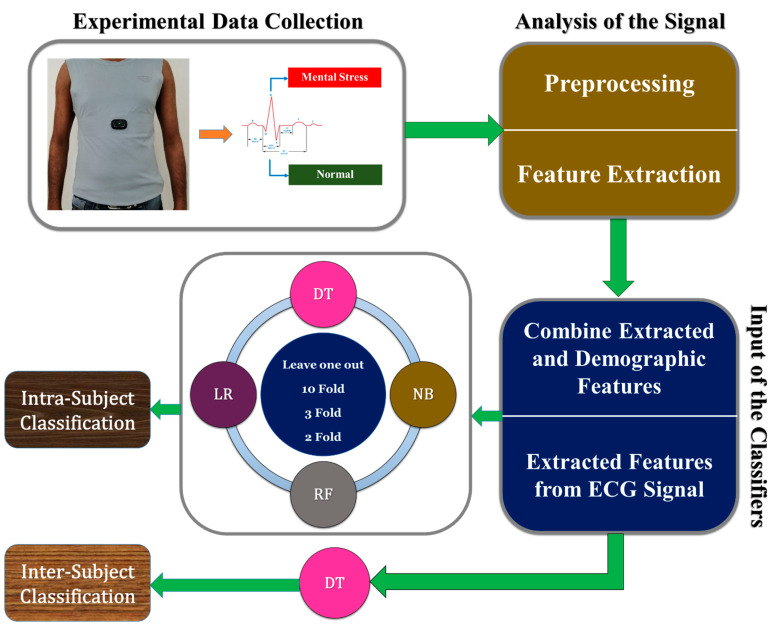
Block diagram of the proposed study.

The proposed study was completed in some steps, such as the selection of the researchers as a subject, recording the ECG signals of the researchers in real-time from 20 subjects (mental stress and normal) using a wearable smart T-shirt, collecting the demographic features, analysis of the ECG signal, extraction of the ECG features, and classification of the intra-subject (mental stress and normal) using DT, NB, RF, and LR machine learning classifiers (Figure 3). In addition, we also used inter-subject classification using the DT machine learning classifier. This study was implemented in MATLAB 2016a and Anaconda3 (64-bit) on Windows 10. 

### 3.1. Experimental Design and Data Collection

For this proposed study, a total of twenty male researchers without mental and heart diseases were recruited from the University of Electronic Science and Technology of China, Chengdu, Sichuan, China (Table 1). Their ages ranged from 23 to 35 years, height from 162 to 187 cm, weight from 48 to 88 Kg, and Body Mass Index (BMI) from 18.289 to 29.068 Kg/m^2^ with no history of medication and sleep disorders. All subjects were asked not to drink alcohol, coffee, or tea for twelve hours before ECG recording. All subjects gave informed consent to the proposed study procedures, and the University Ethical Committee (No: 1061420210210829008) approved the proposed study. Before the experiment, each subject filled the Chalder Fatigue Scale (CFS) [58], Specific Fatigue Scale (SFS) [59], and Depression, Anxiety, and Stress Scale (DASS) [60,61] to measure the mental stress and other mental problems. Previously, the Cold Pressor Stress (CPS) test [62,63,64,65,66], stress determination test, Montreal imaging stress task [67,68], and DASS [60,61] have been used to detect the mental stress.

In the experiment, we used a smart T-shirt to record ECG signals. We choose the 2:1 ratio between mental stress and normal condition. We recorded the mental stress conditions after 12 h of continuous work in the laboratory. We also recorded the normal conditions after waking up in the morning when not working in the laboratory. We recorded the mental stress of the researchers at one-day intervals. This reduces the likelihood of errors in data acquisition.

### 3.2. Preprocessing and Features Calculation

We preprocess the ECG signal using a Fast Fourier Transform (FFT) with a low pass Finite Impulse Response (FIR) filter to remove signal noise. The demographic characteristics are computed during the experiment. Additionally, the other features are calculated during the analysis of the signal. For the demographic features, we calculated age (year), height (cm), weight (Kg), and BMI (Kg/m^2^). 

Heart Rate Variability (HRV) is a set of methodical evaluation indicators obtained from cardiac signals [69]. It reveals information about the variant of RR intervals of the heart [70]. It is measured to reproduce the body’s capacity to adapt or modify the exogenous and endogenous impact on blood supply requirements [71]. We also calculated some features like Average Heart Rate (AHR), Mean of the RR interval (MRR), and HRV features such as Root Mean Square (RMS), Turning Point Rating (TPR), the Standard Deviation of the Heart Rate (SDHR), Root Mean Square Distance of Successive RR interval (RMSSD), several R peaks in ECG that differs more than 50 milliseconds (NN50), Percentage of the Number of R peaks in ECG that differs more than 50 milliseconds (PNN50), and Standard Deviation of the RR interval (SDRR) from ECG signal.

### 3.3. Classification Techniques

For this study, we used five machine learning classifiers such as DT, NB, RF, and LR, with four models such as leave one out, 10-fold, 3-fold, and 2-fold. These classifiers are well defined: DT is a directed learning method used for regression and classification processes. Each outcome of the DT is represented in a separate leaf [72,73,74]. DT has two nodes in its structure: (i) the Decision node, which has multiple branches and is used to make any decision; (ii) the Leaf node, which is the output of the decision nodes without any further branches. DT is easy to understand because it mimics human thinking ability for decision making. The output of the DT algorithm can be easily represented in a tree-like structure. The main challenge of the DT is the identification of the characteristic for the root node at every level, which is called attribute determination. We used a minimum induce binary tree with two number of instances in leaves in our DT classifier. 

NB classifier is based on the Bayes theorem with free expectations between predictors. It is easy to build without difficult iterative parameter estimation, making it individually useful for massive datasets [38]. Bayes theorem provides the calculation of posterior probability from the prior probability of target, the prior probability of predictor, and the probability of predictor given target. NB classifier accepts the effect of the value of a prior probability of predictor on a given target.

RF is an ensemble learning technique for regression, classification, and other works. It constructs a multitude of trees in training and output based on a single tree [75,76]. We used ten number of trees and five number of attributes considered at each split. 

LR is a linear method to make the relationship between the dependent and independent variables. The independent variable event is called simple linear regression. The LR models frequently fit using the least square method. LR was the first type of regression analysis to be used rigorously in real-time applications [77]. 

### 3.4. Performance Evaluation of the Proposed System

We used DT, NB, RF, and LR classifiers with leave one out, 10-fold, 3-fold, and 2-fold models for the intra-subject (mental stress and normal) classification. We also used two inputs, such as nine features including AHR, MRR, and seven HRV, and thirteen features including AHR, MRR, seven HRV, and four demographic features, of the classifiers. In addition, we also classify the inter-subject classification using the DT classifier based on the leave one out model. We obtained some performance measures such as recall, specificity, precision, accuracy, F1, and Area Under the Curve (AUC) [42,43,78,79]. The standard performance measures are described in Equations (1)–(5).

(1) $$recall=\left(\frac{TP}{TP+FN}\right)$$

(2) $$specificity=\left(\frac{TN}{FP+TN}\right)$$

(3) $$precision=\left(\frac{TP}{TP+FP}\right)$$

(4) $$accuracy=\left(\frac{TP+TN}{TP+FP+TN+FN}\right)$$

(5) $$F1=\left(\frac{2\times recall\times precision}{recall+precision}\right)$$

where *TP* is the true positive, *TN* is the true negative, *FP* is the false positive, and *FN* is the false negative.

### 3.5. Normalization Method

The common normalization aspects used for HRV comprise the variance of the R-R interval and the length of the data segment evaluated. The variance is statistically equal to the total power of the RR interval in the time series [44,80]. It is the standard method used before classification to normalize the value of the feature. 

### 3.6. Statistical Analysis

Statistical analysis is very important for the significant difference. We applied the *t*-test to check the significant difference between the model performances [81,82,83,84,85]. We set the value of *p* < 0.05. If the probability is less than 0.05, the difference is significant; otherwise, it is not significant. 

## 4. Results and Discussion

Progressively, most studies indicate that heart problems and related diseases are predicted based on abnormalities in the ECG [86,87]. In general, the corrected QT interval (QTc) is the most important pathological finding in the ECG. Whether a person has a heart problem or not, QTc calculation is used to predict cardiovascular mortality because the QT interval reflects ventricular repolarization and depolarization based on the heartbeat [88,89]. Furthermore, most diseases in which there is an association between QTc prolongation and cardiovascular mortality are due to the fatal ventricular arrhythmias caused by QTc prolongation because of the early development of ventricular depolarization. Some recent studies confirm the association between cardiovascular mortality and QTc prolongation in patients with type 2 diabetes mellitus [90,91].

Mitochondrial dysfunction leads to oxidative stress, which is associated with various pathological processes in many metabolic diseases such as diabetes mellitus, age-related disorders associated with cardiovascular mortality and morbidity, and death [92,93]. Also, in mental stress and mental trauma, mitochondrial dysfunction affects many physiological functions of the endocrine system, nervous system, and immune system, which plays a significant role in allostatic and stress pathophysiology, leading to various types of health problems [94,95,96]. 

Psychological stress generally influences cardiovascular diseases such as coronary heart disease. In 1910, *Obraztsov* and *Strazhesko* first time described that coronary thrombosis and negative emotions could trigger the development of acute myocardial infarction. Schwartz et al. [97] described that terrorist attacks, wars, natural disasters, and financial crises are among the stressful, life-threatening events and positive emotions, such as sports competitions, Christmas and New Year, etc., that have been diagnosed as crucial triggers of cardiovascular risks and related problems in susceptible persons. According to the inter-heart case-control study, negative psychosocial factors also represent variable risk factors associated with adverse cardiovascular prognosis and mortality [98]. De Vente et al. [99] also mentioned that most metabolic syndromes are caused by work-related stress. Significantly, male workers suffering from fatigue and psychological stress have shortened parasympathetic activity, increased sympathetic activity, and decreased Hypothalamic Pituitary Adrenal (HPA) axis response. This work-related stress can also lead to dysregulation of mitochondria and sympathetic-vagal balance. In addition, this type of mental stress stimulates the activity of the sympathetic nervous system and leads to changes in cardiac arrhythmias and a reduction in the occurrence of atrial and ventricular arrhythmias; these variations lead to changes in ECG signals based on the cardiac repolarization phase [100]. So, if a person has cardiovascular problems or has no symptoms, it is recommended to examine his heart functions by ECG and echocardiography to identify and treat the problems and other metabolic disorders as soon as possible [35,101]. Therefore, this study was designed to assess the mental stress of scientific researchers in real-time using ECG signals to evaluate cardiovascular risk factors and associated problems based on machine learning techniques.

### 4.1. Analysis of the Signal

**Figure 4:**
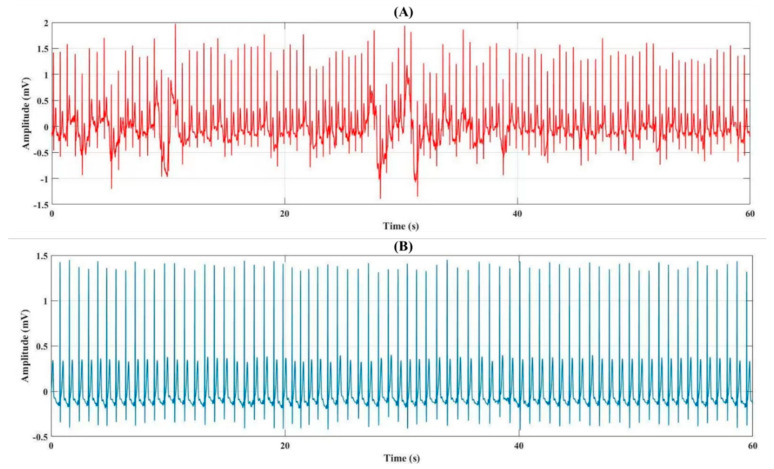
One minute signal representation of the single-lead raw ECG signal from (**A**) mental stress and (**B**) normal subjects.

**Figure 5:**
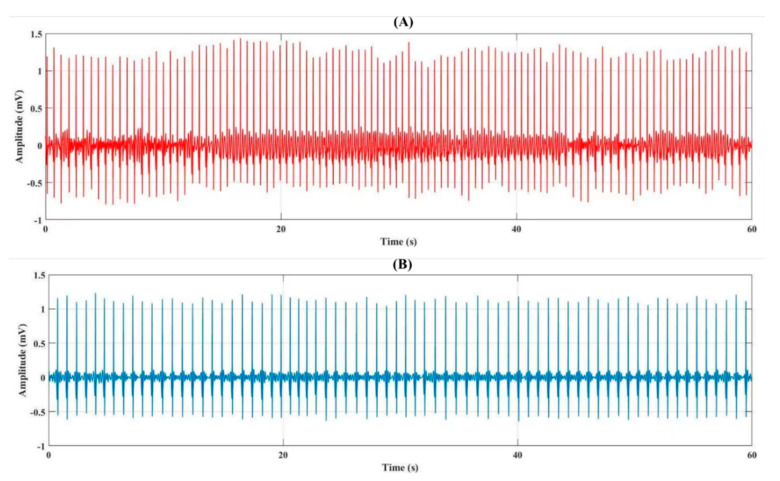
One minute signal representation of the single-lead filtered ECG signal from (**A**) mental stress and (**B**) normal subjects.

We used real-time researchers’ ECG signals in both mental stress and normal conditions (Figure 4). We used 20 subjects of the ECG signal for a total of 1800 min with one-minute data segmentation in this study. Besides, the sampling rate of the ECG signals is 250 Hz. In the preprocessing of the ECG signal, we removed the lower frequencies of the ECG signal using FFT and eliminated the noise of the ECG signal using a low pass FIR filter (Figure 5). The FIR filters are designed by finite-length approximation to an ideal impulse response of infinite-length in the mean square sense obtained by truncation. The number of tabs used is 143. Therefore, the order of the filter is 142. A rectangular window function is used and the cut-off frequency is 50 Hz. 

**Figure 6:**
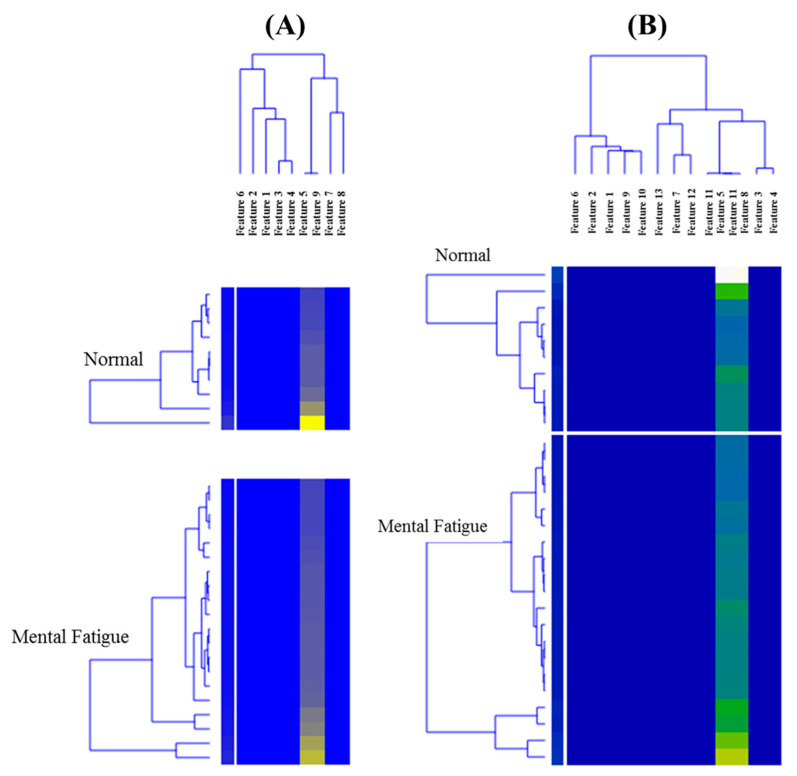
Heat map of the (**A**) AHR, MRR, and seven HRV features, and (**B**) AHR, MRR, seven HRV, and four demographics from mental stress and normal subjects. It showed the relationship between the two features of the subjects, including mental stress and normal.

The advantages of FIR are: (i) they allow maintaining the exact linear phase between input and output signals, and (ii) the filter transfer function always remains stable with quantized coefficients. However, FIR filters suffer from higher computational complexity than their Infinite Impulse Response (IIR) counterparts. Nevertheless, the use of FIR filters should not be a problem unless the designer has a choice between digital signal processors and computers. We designed the relationship of the features using the heat map shown in Figure 6, which is based on clustering techniques. 

### 4.2. Intra-Subject (Mental Stress and Normal) Classification Results of the Proposed System

We used four classifiers such as DT, NB, RF, and LR classifiers with four models: leave one out, 10-fold, 3-fold, and 2-fold for the classification of mental stress and normal subject. Besides, we used two types of inputs for the classifier: (i) AHR, MRR, and seven HRV features, and (ii) AHR, MRR, seven HRV, and four demographic features. 

**Figure 7:**
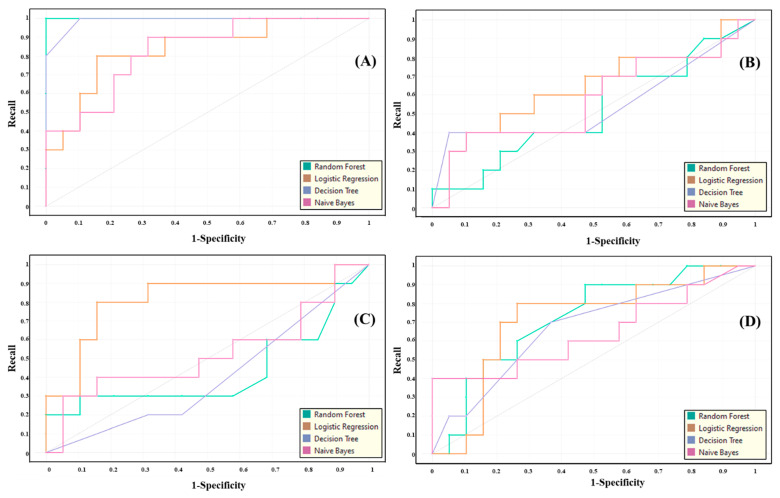
ROC curves of the intra-subject (mental stress and normal) classification with models such as (**A**) leave one out, (**B**) 10-fold, (**C**) 3-fold, and (**D**) 2-fold on nine features extracted by the ECG signal.

The system’s performance using AHR, MRR, and seven HRV features are mentioned in Table 2. The DT leave one out model that achieved the highest performance in terms of recall, specificity, precision, accuracy, and F1, which were found to be 0.933, 0.967, 0.944, 0.933, and 0.935, respectively. The NB 3-fold model achieved the lowest performance in terms of recall, specificity, precision, accuracy, and F1, which were 0.467, 0.333, 0.467, 0.467, and 0.467, respectively. Additionally, the average performance with ±Standard Deviation (SD) in terms of recall, specificity, precision, accuracy, F1, and AUC was found to be 0.664 ± 0.136, 0.597 ± 0.165, 0.679 ± 0.132, 0.664 ± 0.136, 0.661 ± 0.134, and 0.676 ± 0.173, respectively. However, it indicates that the LR classifier has good performances in three models such as 10-fold, 3-fold, and 2-fold. In addition, the NB classifier has poor performances in all models. The Receiver Operating Characteristics (ROC) curve of the models using AHR, MRR, and seven HRV features is mentioned in Figure 7.

**Figure 8:**
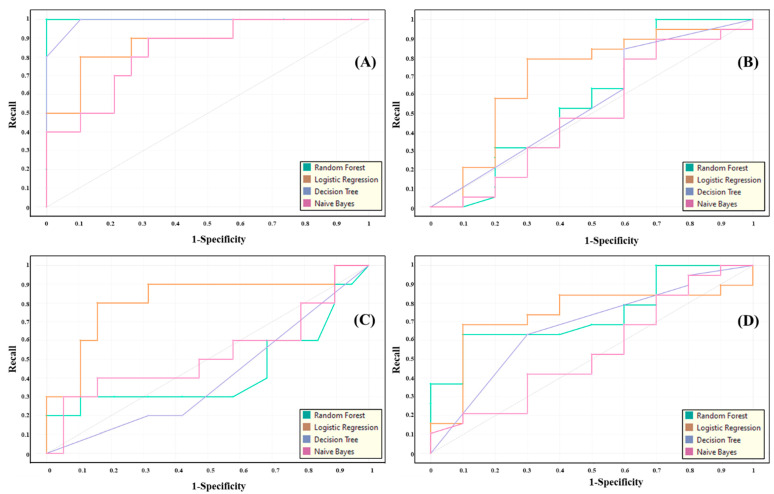
ROC curves of the intra-subject (mental stress and normal) classification with models such as (**A**) leave one out, (**B**) 10-fold, (**C**) 3-fold, and (**D**) 2-fold on thirteen features, including demographic and extracted through ECG signal.

The performance of the system using AHR, MRR, seven HRV, and four demographic features are mentioned in Table 3. The DT leave one out model achieved the highest performance in terms of recall, specificity, precision, accuracy, and F1, which were found to be 0.933, 0.967, 0.944, 0.933, and 0.935, respectively. The NB 10-fold model achieved the lowest performance in terms of recall, specificity, precision, accuracy, and F1, which were 0.400, 0.450, 0.481, 0.400, and 0.411, respectively. Additionally, the average performance with ± SD in terms of recall, specificity, precision, accuracy, F1, and AUC was found to be 0.656 ± 0.148, 0.603 ± 0.166, 0.677 ± 0.138, 0.656 ± 0.148, 0.654 ± 0.145, and 0.673 ± 0.185, respectively. However, it indicates that the LR classifier has good performances in three models such as 10-fold, 3-fold, and 2-fold. In addition, the NB classifier has poor performances in all models. The ROC curve of the models using AHR, MRR, seven HRV, and four demographic features are shown in Figure 8.

### 4.3. Performance of the Inter-Subject Classification Using DT Classifier 

We found that the DT classifier for the leave one out model based on both inputs had maximum accuracy in the system. However, we applied the DT classifier for the inter-subject classification mentioned in Table 4. The average performance (inter-subject) measures in terms of recall, specificity, precision, accuracy, F1, and AUC was found to be 0.938, 0.964, 0.947, 0.941, 0.939, and 0.990, respectively. 

### 4.4. Comparison between Proposed and Previously Selected Methods

**Figure 9:**
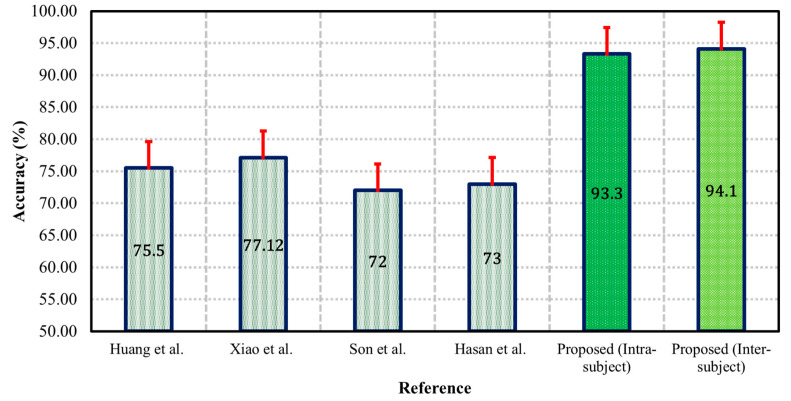
Comparison between previously published and proposed accuracy of the system.

Machine learning technique is the one type of application of artificial intelligence. It is used in the different areas of human research, from early prediction to treatment. In mental stress, we have used DT, NB, RF, and LR machine learning classifiers to detect the researchers’ mental stress. Researchers are an essential part of modern societies. Without their help, it is impossible to obtain emerging technology. We used a wearable smart T-shirt to record the researchers’ ECG signals, and this device can easily record the signals without any problem. Previously, many researchers have used different wearable smart devices to record the physiological signals to detect the mental stress of the assembly-line operators, brain-injured patients, drivers, equipment operators, pilots, security guards, and traffic controllers [28,31,102,103,104,105]. They employed various techniques to find a more accurate detection system using wearable smart devices such as SYMTOP NT9200, NI USB-6008, ST-BTA, Storm 3G Ranger X, ADS1292R, Emotiv EPOC, and HD-BTA (Table 5). Various kinds of stress may facilitate atrial and ventricular arrhythmias. Evaluation of ECG signals related to stress symptoms may provide systematic evidence for further studies to improve cardiovascular and related therapy. Our analysis achieved the maximum accuracy (intra-subject classification: 93.30% and inter-subject classification: 94.10%) compared with the previous mental stress detection system based on intra-subject (mental stress vs. normal) and inter-subject classifications (Figure 9). 

### 4.5. Applications and Limitations of the Proposed Study

The proposed work shows the application for researchers’ mental stress using a wearable smart T-shirt. The DT and LR machine learning classifiers easily classify both mental stress and normal conditions using ECG signals. The proposed system would provide a more accurate, reliable, and efficient automatic system for detecting mental stress. It is used in the assessment of sleep quality, prediction of the Sudden Cardiac Death (SCD), detection of the heartbeat, sleep disorder, premature beat, mental stress of human beings, and associated metabolic syndromes [45,108,109,110,111,112]. This system will help doctors save harmful diseases and increase the efficiency of their work. 

The proposed work has some limitations, such as gender equality, the number of subjects, and only one psychological signal, because it is difficult to find volunteers for the study in the given time and also in the current COVID-19 epidemic policy. We also discussed mental stress and its association with mitochondrial dysfunction leading to cardiovascular diseases such as atrial and ventricular arrhythmias and associated problems to illustrate the importance of this study. In addition, there is the possibility of over-fitting all models due to the limited sample size, the fact that the subjects are relatively young and healthy, and that this is a group of scientific researchers different from a group of truck drivers, etc. 

## 5. Conclusions

Mental stress is prevalent in the modern world and is related to reduce performance on cognitive tasks. This proposed study has developed an automatic detection system for the researchers’ mental stress using an ECG signal recorded by a wearable smart T-shirt. The obtained results show that the DT classifier achieved the highest accuracy (93.30%) for intra-subject classification. In addition, the inter-subject classification of the DT classifier has a 94.10% average accuracy. This system is more efficient and easier to monitor the cardiac signal, especially for the researchers in this study. To the best of our knowledge, the proposed system is better than the previously selected systems using wearable smart devices. In the future, an automatic detection system for psycho-neurological human behaviors based on mitochondrial dysfunction and anxiety using physiological signals could be planned to save researchers from harmful diseases with a large number of subjects. 

## Figures and Tables

**Table 1:** Description of the experimental dataset.

Total No. of the Subjects (N)	20
Occupation of the Subjects	Research (Ph.D. and Research Associate)
Conditions of the Subjects	Mental Stress and Normal
Gender of the Subjects	All are Male
The total duration of the recordings (T)	1800 min
The total duration of the mental stress recordings (T_MF_)	1200 min
The total duration of the normal recordings (T_N_)	600 min

**Table 2:** Performance of the intra-subject (mental stress and normal) classification using nine features, including AHR, MRR, and seven HRV features.

Model	Classifier	Recall	Specificity	Precision	Accuracy	F1	AUC
**Leave one out**	**DT**	**0.933**	**0.967**	**0.944**	**0.933**	**0.935**	0.990
	NB	0.733	0.817	0.807	0.733	0.741	0.840
	RF	0.900	0.800	0.913	0.900	0.895	1.000
	LR	0.800	0.700	0.795	0.800	0.794	0.835
10-fold	DT	0.567	0.483	0.577	0.567	0.571	0.540
	NB	0.533	0.467	0.556	0.533	0.542	0.570
	RF	0.633	0.467	0.607	0.633	0.614	0.530
	LR	0.700	0.550	0.683	**0.700**	0.684	0.630
3-fold	DT	0.467	0.483	0.533	0.467	0.481	0.530
	NB	0.467	0.333	0.467	0.467	0.467	0.410
	RF	0.633	0.467	0.607	0.633	0.614	0.435
	LR	0.767	0.683	0.762	**0.767**	0.763	0.815
2-fold	DT	0.667	0.433	0.628	0.667	0.617	0.680
	NB	0.500	0.550	0.579	0.500	0.512	0.633
	RF	0.633	0.667	0.689	0.633	0.644	0.692
	LR	0.700	0.700	0.729	**0.700**	0.707	0.700
Average		0.664	0.597	0.679	**0.664**	0.661	0.676
±Standard Deviation		0.136	0.165	0.132	**0.136**	0.134	0.173

**Table 3:** Performance of the intra-subject (mental stress and normal) classification using thirteen features, including AHR, MRR, seven HRV, and four demographic features.

Model	Classifier	Recall	Specificity	Precision	Accuracy	F1	AUC
Leave one out	DT	**0.933**	**0.967**	**0.944**	**0.933**	**0.935**	0.990
	NB	0.733	0.817	0.807	0.733	0.741	0.840
	RF	0.867	0.733	0.889	0.867	0.856	1.000
	LR	0.867	0.833	0.867	0.867	0.867	0.880
10-fold	DT	0.567	0.483	0.577	0.567	0.571	0.550
	NB	0.467	0.433	0.512	0.467	0.481	0.495
	RF	0.633	0.467	0.607	0.633	0.614	0.573
	LR	0.733	0.617	0.723	**0.733**	0.725	0.685
3-fold	DT	0.500	0.400	0.512	0.500	0.505	0.438
	NB	0.400	0.450	0.481	0.400	0.411	0.440
	RF	0.567	0.433	0.556	0.567	0.561	0.418
	LR	0.733	0.667	0.733	**0.733**	0.733	0.790
2-fold	DT	0.667	0.433	0.628	0.667	0.617	0.680
	NB	0.500	0.600	0.608	0.500	0.505	0.537
	RF	0.633	0.617	0.664	0.633	0.642	0.737
	LR	0.700	0.700	0.729	**0.700**	0.707	0.730
Average		0.656	0.603	0.677	**0.656**	0.654	0.673
±Standard Deviation		0.148	0.166	0.138	**0.148**	0.145	0.185

**Table 4:** Performance of the inter-subject (subject-wise) classification using DT classifier for the leave leave-one-out model.

Subject	Recall	Specificity	Precision	Accuracy	F1	AUC
Normal 1	0.966	0.984	0.969	0.966	0.966	0.997
Normal 2	0.966	0.984	0.969	0.966	0.966	0.994
Normal 3	0.931	0.969	0.944	0.931	0.933	0.989
Normal 4	0.931	0.969	0.944	0.931	0.933	0.989
Normal 5	0.931	0.969	0.944	0.931	0.933	0.989
Normal 6	0.931	0.969	0.944	0.931	0.933	0.989
Normal 7	0.931	0.969	0.944	0.931	0.933	0.989
Normal 8	0.966	0.984	0.969	0.966	0.966	0.994
Normal 9	0.931	0.969	0.944	0.931	0.933	0.989
Normal 10	0.931	0.969	0.944	0.931	0.933	0.989
Mental Stress 1	0.964	0.980	0.968	0.964	0.965	0.997
Mental Stress 2	0.929	0.960	0.940	0.989	0.930	0.989
Mental Stress 3	0.929	0.916	0.929	0.929	0.929	0.986
Mental Stress 4	0.929	0.960	0.940	0.929	0.930	0.989
Mental Stress 5	0.929	0.916	0.929	0.929	0.929	0.986
Mental Stress 6	0.929	0.960	0.940	0.929	0.930	0.989
Mental Stress 7	0.929	0.960	0.940	0.929	0.930	0.989
Mental Stress 8	0.929	0.960	0.940	0.929	0.930	0.986
Mental Stress 9	0.929	0.960	0.940	0.929	0.930	0.989
Mental Stress 10	0.964	0.980	0.968	0.964	0.965	0.997
Average	**0.938**	**0.964**	**0.947**	**0.941**	**0.939**	**0.990**
±Standard Deviation	0.015	0.018	0.012	0.018	0.014	0.003

**Table 5:** Comparison between previously selected and proposed methods.

Reference	Year	Subject	Recording Device	Signal	Accuracy
Huang et al. [105]	2018	35	ADS1292R	ECG	75.50
Xiao et al. [31]	2018	5	Emotiv EPOC	EEG	77.12
Son et al. [106]	2018	32	AgCl Electrodes	EEG, EOG, EMG	72.00
Hasan et al. [107]	2019			EEG	73.00
**Proposed (Intra-subject)**		**20**	**Smart T-Shirt**	**ECG**	**93.30**
**Proposed (Inter-subject)**					**94.10**

## Data Availability

As per the request through the Dr. Md Belal Bin Heyat (belalheyat@gmail.com).
